# Curcumin-loaded nanoemulsion for acute lung injury treatment *via* nebulization: Formulation, optimization and *in vivo* studies

**DOI:** 10.5599/admet.2661

**Published:** 2025-03-20

**Authors:** Prashant Anilkumar Singh, Rajendra Awasthi, Ramendra Pati Pandey, Santosh K. Kar

**Affiliations:** 1 Department of Allied Sciences, School of Health Sciences and Technology, UPES, Dehradun-248 007, Uttarakhand, India; 2 Department of Pharmaceutical Sciences, School of Health Sciences and Technology, UPES, Dehradun-248 007, Uttarakhand, India; 3 Department of Biotechnology, SRM University, Delhi-NCR, Sonepat-131029, Haryana, India; 4 Polyfeenolix Research Laboratory (OPC) Private Limited, Patia, Bhubaneswar-751024, Odisha, India

**Keywords:** Acute lung injury, acute respiratory distress syndrome, nanoemulsion, nebulization, pulmonary delivery

## Abstract

**Introduction:**

Curcumin, a polyphenolic bioactive molecule, exhibits potent anti-inflammatory and antioxidant properties by reducing cytokine levels such as IL-6, TNF-α, and TGF-β. It regulates IL-17A and modulates key signaling pathways, including PI3K/AKT/mTOR, NF-κB and JAK/STAT. However, its clinical application is hindered by rapid metabolism, poor solubility, and chemical instability.

**Method:**

Using the Box-Behnken design, this study developed and optimized a curcumin-loaded turmeric oil-based nanoemulsion system. The effects of turmeric oil, Tween 80 and sonication cycles on particle size (PS), polydispersity index (PDI), and encapsulation efficiency were analyzed. The optimized nanoemulsion was characterized by zeta potential, PDI, PS, morphology, loading efficiency, EE, and antioxidant activity (DPPH assay). *In vitro* cytotoxicity was evaluated using A549 cells, while *in vivo* efficacy was assessed in BALB/c mice through histological analysis, bronchoalveolar lavage fluid analysis, and TNF-α and IL-1β estimation via enzyme-linked immunosorbent assay.

**Results:**

The optimized nanoemulsion had high entrapment efficiency (92.45±2.4 %), a PS of 130.6 nm, a PDI of 0.151, and a zeta potential of -1.7±0.6 mV. Nanoparticle tracking analysis confirmed a mean PS of 138.3±1.6 nm with a concentration of 3.78×10^12^ particles/mL. Transmission electron microscopy imaging confirmed spherical morphology. The *IC*_50_ value was 25.65 μg/mL. The nanoemulsion remained stable for three months at 4±1 and 25±2 °C/ 60±5 % relative humidity. The optimized formulation significantly reduced BALF total cell count, alveolar wall thickening, and TNF-α and IL-1β levels (*p* < 0.001).

**Conclusion:**

Overall, the optimized formulation significantly lowered levels of pro-inflammatory cytokines in the acute lung injury /acute respiratory distress syndrome mouse model.

## Introduction

Acute lung injury (ALI) is a life-threatening pulmonary condition that is characterized by alveolar damage, pulmonary edema, inflammatory infiltration, and microvascular permeability. These characteristics further contribute to acute hypoxia and respiratory dysfunction [1-3]. ALI affects 16 out of 100,000 and 306 out of 100,000 individuals aged between 15 and 19 and 75 and 84, respectively [4]. ALI can be caused by several factors, including trauma, blood transfusion, chemicals, diseases like COVID-19, pancreatitis, drug toxicity, infection, inhalation injuries, and amniotic fluid embolism. If ALI is not treated in its early stages, it can progress to acute respiratory distress syndrome (ARDS) [5,6]. In addition, many current therapeutic strategies, such as ventilation, corticosteroids, vasodilator drugs, and antimicrobial therapy, have been used for ALI and ARDS treatment. However, undesirable side effects of drugs and the complex pathophysiology of ALI hinder their treatment approaches [7,8]. The preclinical model of ALI or ARDS can be developed by administering a single dose of lipopolysaccharide (LPS) via the intranasal or intratracheal routes. This model exhibits similar features of human ARDS, such as capillary barrier permeation, neutrophil infiltration, inflammatory response, lung damage, and difficulty in breathing [9,10].

Curcumin is a yellow-colored polyphenolic bioactive molecule present in the rhizome of *Curcuma longa* of the *Zingiberaceae* family. It is a well-known molecule for its numerous therapeutic activities, such as antioxidant, anti-inflammatory, anti-cancer, anti-bacterial, and anti-viral. It also provides neuroprotection, immunomodulation, and cardiovascular protection [11,12]. It also has effective therapeutic outcomes against respiratory disorders like ALI, chronic obstructive pulmonary disease, pulmonary fibrosis, and asthma. Curcumin has been documented to suppress cytokine levels such as TNF-α, IL-6, and TGF-β and can regulate IL-17A [13,14]. Curcumin can regulate the NF-κB (nuclear factor kappa-B), JAK/STAT (Janus kinase/signal transducer and activator of transcription), and PI3K/AKT/mTOR (phosphoinositide-3-kinase/protein kinase B/mechanistic target of rapamycin) pathways, which are involved in ALI. However, curcumin’s poor solubility, rapid metabolism, unfavorable pharmacokinetic features, and chemical instability hinder its clinical application [12,15,16].

Nanoemulsions are colloidal systems that form when immiscible liquids become miscible with the aid of surfactants. The surfactant absorbs between the oil/water surface and reduces surface tension, preventing emulsion's coalescence. They are kinetically stable and thermodynamically unstable. They can encapsulate both hydrophilic and hydrophobic drugs [17,18]. They also enhance the bioavailability of drug molecules. Nanoemulsion can deliver the drugs to the pulmonary site with good lung distribution. Nanoemulsions provide protection to drug molecules from enzymatic degradation, light, oxidation, and temperature [19,20]. Nebulization is a method that offers direct administration of therapeutic molecules in fine mist or aerosol form to the lungs by using nebulizers. It does not require coordination between patient and actuator and is easy to operate [17,21].

The aim of this research was to develop a curcumin-loaded nanoemulsion for ALI treatment via a nebulization approach. We formulated the curcumin nanoemulsion using the ultrasonication method and optimized it using the Box-Behnken design. The optimized formulation was subjected to *in vitro* evaluation like encapsulation efficiency (EE), polydispersity index (PDI), zeta potential, drug loading (DL), particle size (PS), transmission electron microscopy (TEM), *in vitro* release, stability study, antioxidant activity and nitric oxide analysis. The optimized formulation was also subjected to determine its therapeutic efficacy against the LPS-induced ALI model in BALB/c mice.

## Experimental

### Materials

Curcumin, Tween 80, and ethanol were purchased from Sigma Aldrich, MA, United States. Turmeric oil was a kind of gift sample received from Polyfeeenolix Research Laboratory Pvt. Ltd., Orissa, India. Formalin was purchased from SRL Chemicals (Mumbai, India). ELISA kit was purchased from R&D Systems, Inc. (Minneapolis, United States). Nebulizer NE 100, Ross Max therapy was purchased from Rossmax Swiss GmBH, (Heerbrugg, Switzerland). LPS (strain O111:B4) was procured from Merck, MA, United States.

### Preparation of curcumin-loaded nanoemulsion

Curcumin-loaded oil-in-water (o/w) nanoemulsion was developed using the high-energy ultrasonication method. Turmeric oil was used as the oil phase, Tween 80 was used as a surfactant, and 0.9 % saline was used as an aqueous phase. Curcumin was added to the mixture of turmeric oil and Tween 80 and vortexed to form a lipid phase (Table 1). Saline solution (0.9 %) was added into the lipid phase and vortexed to obtain the product containing 100 μg/mL curcumin. The resultant mixture was sonicated at a high intensity (40 % amplitude and 22 kHz frequency with 400 W power) with a pulse rate of 20 s on/off at 50 °C to obtain the final nanoemulsion formulation [22].

**Table 1.** Box-Behnken design used to formulate curcumin-loaded nanoemulsion

Formulation	Independent variables		
	*A*: Turmeric oil amount	*B*: Tween 80 amount	*C*: Sonication cycle time
CNE_1_	0	1	1
CNE_2_	-1	-1	0
CNE_3_	-1	0	1
CNE_4_	1	1	0
CNE_5_	1	0	1
CNE_6_	1	-1	0
CNE_7_	0	0	0
CNE_8_	0	-1	-1
CNE_9_	-1	0	-1
CNE_10_	-1	1	0
CNE_11_	1	0	-1
CNE_12_	0	1	-1
CNE_13_	0	-1	1
Codes for different independent variables	Values for independent variables		
	Turmeric oil amount, %	Tween 80 amount, %	Sonication cycle, min
-1	0.5	0.1	6
0	1.0	0.15	8
+1	1.5	0.2	10
Dependent variables			
*Y*_1_ = PS	Minimum		
*Y*_2_ = PDI	Minimum		
*Y*_3_ = EE	Maximum		

### Experimental design for the optimization of curcumin-loaded turmeric oil-based nanoemulsion

The nanoemulsion optimization was performed using the Box-Behnken design in the DOE software (V10.0.4, Stat-Ease, Minneapolis, USA). Independent variables were turmeric oil (*A*), Tween 80 (*B*), and sonication cycle (*C*), and the dependent variables were PS, PDI, and EE (Table 1). We optimized the effect of independent variables on dependent variables using a 3-level factorial design. Statistical tests, including regression coefficient (*R*^2^), goodness-of-fit, and analysis of variance (ANOVA) were used to ascertain the relevance of the developed model.

### Mathematical model

Design Expert v23.0.0 64-bit software (Stat-Ease, Inc., Minneapolis, MN) was used to precisely investigate the effects of independent variables on dependent variables. This software facilitated the creation of a nonlinear quadratic polynomial model. To optimize the nanoemulsion formulations using the quadratic model (Equation 1), when evaluating the main effect of independent variables, the significance of the center point region of the three-dimensional cube was determined.



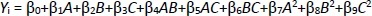

(1)


Where *Y*_i_ represents dependent variables corresponding to each factor, *A* represents the effect of turmeric oil, *B* represents the effect of Tween 80, and *C* represents the sonication cycle period. Independent variables (*A*, *B*, and *C*) were considered at their low (-1), median (0), and high (+1) values. The effect of turmeric oil was measured at three levels: the low level was 0.5 %, the median level was 1 %, and the high level was 1.5 %. The effect of Tween 80 was measured at 0.1 % (low), 0.15 % (median), and 0.2 % (high) levels. In addition, the last independent variable was the sonication cycles that were considered 6, 8, and 10 min from low to high values (Table 1).

### Characterization of curcumin-loaded turmeric oil-based nanoemulsion

#### Determination of particle size and polydispersity index

Malvern Nano ZS Zetasizer (Malvern, UK) was used to measure the particle size (PS) for the nanoemulsion and PDI. Before measurement, samples were diluted with distilled water. Due to Brownian motion, the particle change in light scattering was analyzed by the instrument. The instrument conducted an average of 10 measurements at a temperature of 25 °C and a scattering angle of 90°.

#### Nanoparticle tracking analysis

Nanoparticle tracking analysis (NTA) was done to examine PS distribution and measure particle concentration. The analysis was performed with a NanoSight NS300 instrument (Malvern Pananalytical, Malvern, UK) equipped with a sCMOS camera and a 480 nm laser light source for video recording. A microscope with 20x magnification provided an observation field of approximately 100N80810 μm. To enable optimal particle tracking, we diluted the sample to 1:10,000 using ultrapure water prior to analysis. The diluted sample was drawn into a 1 mL syringe and placed in the NTA sample chamber. Three video recordings, each lasting for 60 seconds, were captured for analysis. All measurements were performed under controlled temperature (28.5 to 28.7 °C). Data analysis was done using NTA 3.1 software (Malvern Pananalytical, Malvern, UK) [23].

#### Determination of zeta potential

The Zeta potential of the diluted optimized nanoemulsion (formulation CNE_7_) was determined using Nano ZS Malvern Zetasizer (Malvern, UK). A disposable cuvette (DTS 1060) was used to measure the charge on the emulsion droplet [24].

#### Morphological characterization

The morphological characteristics and the PS of the optimized formulation were examined using high-resolution transmission electron microscopy (TEM) (JEM 2100 Plus, JEOL, Japan) at 80 kV. A drop of diluted nanoemulsion was placed on the carbon-coated copper grid, air-dried, and further stained with 1 % phosphotungstic acid (PTA). The sample was allowed to dry at ambient temperature before imaging [25].

#### Encapsulation efficiency and loading efficiency

The EE and DL of curcumin-loaded nanoemulsion were determined using a centrifugation machine (REMI C-24 PLUS, Mumbai, India). Five milliliters of nanoemulsion was centrifuged at 15000 rpm for 15 minutes at 4 °C. One milliliter syringe was used to collect the unentrapped drug from the aqueous phase of the centrifuge tube. The collected sample was filtered via a 0.22 μm syringe to remove oil droplets. The filtrate was scanned at 425 nm using a UV spectrophotometer (UV-1900 Shimadzu, Japan) to assess the amount of free curcumin. The EE and DL were calculated by using Equations (2) and (3) [26]:



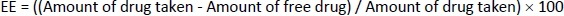

(2)




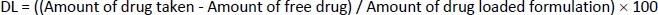

(3)


#### Stability study

The optimized nanoemulsion was stored at 4±1 °C and 25±2 °C / 60±5 % RH for 3 months and examined in terms of for any instability, such as phase separation, sedimentation, PS, PDI, and zeta potential [27].

### In vitro cytotoxicity

#### Cell line and cell culture medium

The human lung adenocarcinoma epithelial cell line (A549) was procured from the National Center for Cell Science, Pune, Maharashtra, India. Cells were maintained in Dulbecco's Modified Eagle Medium (DMEM) supplemented with 10 % heat-inactivated fetal bovine serum (FBS) (Himedia-RM 10432) and 1 % antibiotic solution consisting of Penicillin-Streptomycin cocktail (Sigma-Aldrich P0781). Cultures were incubated at 37 °C in a humidified atmosphere with 5 % CO_2_. Cell detachment was performed using Trypsin-EDTA solution (0.25 % trypsin and 0.02 % EDTA in phosphate buffer saline), and the detached cells were neutralized using DMEM containing 10 % FBS. Post-neutralization, the cells were reseeded in T25 culture flasks and monitored for confluency.

#### Estimation of reactive nitrogen intermediate

On a 96-well plate, 10 μL of the sample or standard were aliquoted into the defined wells. Griess reagent (100 μL) was added to the wells of the control and test, and the plate was incubated for 5 to 10 min at room temperature. The sodium nitrite standard was prepared in methanol/water (50:50 v/v) at a concentration range of 0 to 50 μg/mL. Absorbance was measured at 540 nm using a microplate reader.

#### Antioxidant activity

The *IC*_50_ value is the concentration of sample required to scavenge 50 % of the DPPH radicals. The free radical scavenging activity of the optimized formulation was evaluated using the DPPH assay. In a 96-well plate, 5 μL of different concentrations of the test compound were mixed with 0.1 mL of a DPPH solution prepared in 80 % ethanol. The plate was incubated in the dark for 30 minutes at 27 ± 2 °C. After incubation, the absorbance (Abs) was measured at 517 nm using a microplate reader (iMark, Bio-Rad, CA, United States). The scavenging activity was calculated using Equation (4) [28]:



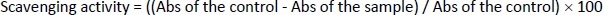

(4)


### In-vivo studies

#### Experimental animals

BALB/c mice, eight to nine weeks old (16-18 g), were procured from the National Institute of Biologics (NIB), Noida, India, and acclimatized for a week. Animals were maintained in a 12-h light and 12-h dark cycle at 22-25 °C with 50 to 60 % humidity. Food and water were given ad libitum.

#### Experimental design

Mice were anesthetized with a 2 % isoflurane solution. While anesthetized, mice received 50 μg/50 μL of LPS dissolved in saline *via intranasal* (*i.n*.) administration to the mice through the nostril. The LPS was applied to the nares with a 20 μL tip using an Eppendorf pipette. The animals were divided into five groups (*n* = 6). Group 1 (control group) received 50 μL of 0.9 % saline *via i.n*. route. Animals of group 2 (negative control group) received 50 μL of *i.n.* LPS. Group 3 (positive control group) animals received 50 μL *i.n.* LPS and 5 mg/kg of dexamethasone via intraperitoneal injection. Group 4 (vehicle control group) animals received 50 μL *i.n.* LPS and nebulized formulation without curcumin. Group 5 (treatment group) animals received 50 μL *i.n.* LPS and optimized formulation (CNE_7_) equivalent to 100 μg/mL of curcumin. After 24 hours of treatment, three mice from each group were sacrificed for BALF analysis and cytokine estimation. Another three mice were sacrificed 48 hours later for histological analysis.

#### Histological analysis

The left lung was harvested from the mice for histopathological examination through a midline incision from the sternum to the diaphragm. Lungs were initially perfused with phosphate buffer saline (PBS) via the left ventricle with a 20G needle. Following cardiac perfusion, the left lung was harvested, preserved in 10 % neutral buffered formalin, and fixed in paraffin. Sections of 5 μm thickness were prepared and stained with hematoxylin and eosin (H&E) for architectural analysis, along with Masson's trichrome staining to assess lung collagen deposition [29].

#### Bronchoalveolar lavages fluid

Bronchoalveolar lavage fluid (BALF) was collected in triplicate from the lungs through intratracheal injection of 1 mL of cold PBS flushed into the lungs and withdrawn. After aspiration, the lavage fluid was centrifuged at 500*g* at 4 °C for 5 min. After centrifugation, the supernatant was collected and stored at -80 °C for ELISA. The pellet obtained was resuspended in 100 μL of PBS, from which 10 μL was subjected to cell count on a hemocytometer. The supernatant of the BAL was used for the total protein content estimation using the Bradford BSA method [30,31].

#### Estimation of TNF-α and IL-1β by ELISA test

Cytokine levels for TNF-α and IL-1β were measured in the BALF supernatant by the ELISA kit according to the manufacturer’s instructions (ELISA kit Duoset, R&D system).

### Statistics

The statistical analysis was performed using GraphPad Prism 8.0.2 (trial version) software. One-way ANOVA, together with Tukey’s post hoc test, was conducted for group comparisons involving three or more groups. Statistical significance was considered at *p* < 0.05. All values were represented as mean ± standard error of the mean (SEM).

## Results and discussion

### Optimization studies

Curcumin-loaded nanoemulsion was developed using ultrasonication and optimization was performed using Box-Behn design in Design Expert® software. Three independent variables (*A*, *B*, and *C*) and three dependent variables (*Y*_1_ - PS, *Y*_2_ - PDI and *Y*_3_ - EE) were selected for framing significant models (Table 1). The interaction effect of independent variables on dependent responses (*Y*_1_, *Y*_2_ and *Y*_3_) was examined (Table 1). Quadratic polynomial models were found to be the most appropriate option to represent and analyze all three factors suitably. The response analysis was performed using ANOVA to find the best-optimized parameters and best mathematical model with the help of ‘*p*-value’ and regression coefficient (*R*^2^). Formulation CNE_7_ (8 minutes sonication time, 1 % turmeric oil and 0.15 % Tween 80) was found to be an optimized formulation (Table 2).

**Table 2.** Results of curcumin-loaded turmeric oil-based nanoemulsion

Formulation	Particle size, nm	Polydispersity index	Encapsulation efficiency, %
CNE_1_	153.0	0.228	81.49 ±1.2
CNE_2_	162.7	0.236	77.91 ± 1.4
CNE_3_	141.9	0.245	84.46 ± 1.8
CNE_4_	170.8	0.188	75.2 ± 2.1
CNE_5_	222.2	0.196	72.38 ± 1.2
CNE_6_	255.4	0.175	74.32 ± 1.7
CNE_7_	130.6	0.151	92.45 ± 2.4
CNE_8_	191.4	0.209	73.62 ± 2.2
CNE_9_	213.3	0.163	86.24 ± 1.5
CNE_10_	182.2	0.198	87.21 ± 2.7
CNE_11_	210.8	0.154	75.42 ± 2.1
CNE_12_	181.9	0.185	82.65 ± 1.1
CNE_13_	204.8	0.288	71.24 ± 2.5

**Figure 1.**
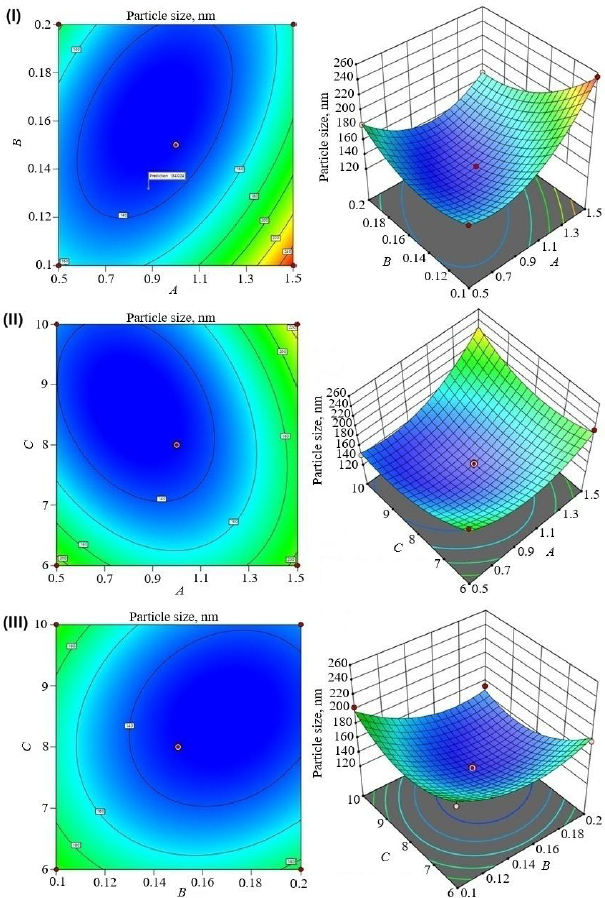
3D response surface and contour plots of PS. (I) effect of turmeric oil and Tween 80, (II) effect of turmeric oil and sonication cycle, and (III) effect of Tween 80 and sonication time

**Figure 2.**
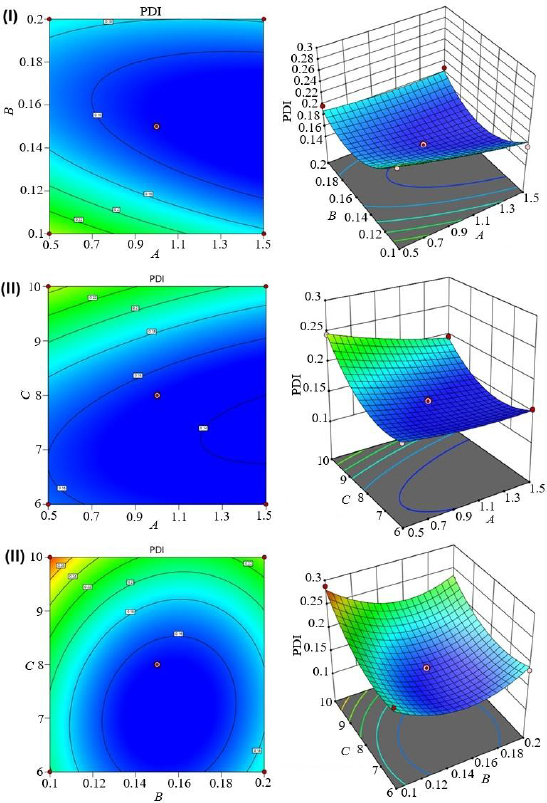
3D response surface and contour plots of PDI. (I) effect of turmeric oil and Tween 80, (II) effect of turmeric oil and sonication cycle, and (III) effect of Tween 80 and sonication time

**Figure 3.**
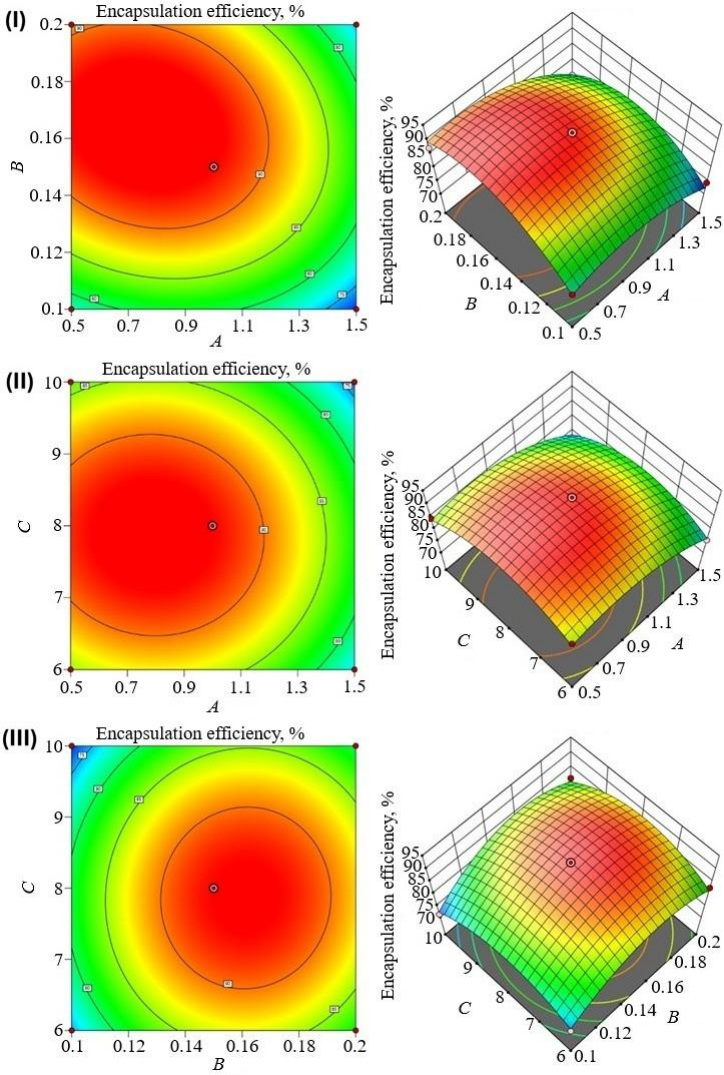
3D response surface and contour plots of EE: (I) effect of turmeric oil and Tween 80 (II) effect of turmeric oil and sonication cycle, (III) effect of Tween 80 and sonication time

Table 2 shows the experimental design runs and their impact on dependent variables. The contour plots and 3D response plots are shown in Figures 1, 2, and 3 as a function of formulation variables PS, PDI, and EE. The relationship between independent and dependent variables was depicted by quadratic equations (Equations (5) to (7)).



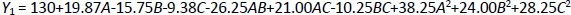

(5)




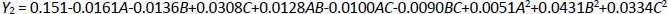

(6)




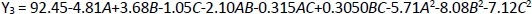

(7)


Where *A* is the effect of turmeric oil, *B* is the effect of Tween 80, *C* is the sonication time, *AB* is the effect of turmeric oil and Tween 80, *AC* is the effect of turmeric oil and sonication time, and *BC* is the effect of Tween 80 and sonication time.

The PS of curcumin-loaded nanoemulsion formulations was found to be 130.6 to 255.4 nm. The positive magnitude of turmeric oil increased the PS of nanoemulsion while the negative magnitude of Tween 80 decreased it (Eq. 1). The response surface plots further elaborated on the relationship between the independent and dependent variables [32,33]. Figure 1 depicts that a higher oil level increases PS because the lower surfactant concentration cannot cover the oil droplets and lowers interfacial tension at the oil/water interface, resulting in larger globules due to coalescence. On the other hand, the high concentration of surfactant resulted in decreased interfacial tension and oil solubilization, which were responsible for the reduction in PS [32-34]. In addition, higher sonication time reduced the PS because the prolonged exposure to ultrasonic energy increases the disintegration of larger droplets into smaller droplets [35]. Turmeric oil (*A*) (*F*-value of 37.00) and Tween 80 (*B*) (*F*-value of 23.23) had a significant effect (*p* < 0.05) on the PS (*Y*_1_). Moreover, sonication time had a negative influence on PS. The PS was significantly (*p*-value of 0.0108 and *F*-value of 32.27) influenced by the combination of turmeric oil and Tween 80 (AB) (Table 3).

**Table 3.** Results of a one-way analysis of variance (ANOVA) of curcumin-loaded nanoemulsion for PS, PDI and EE

Response	Source	Sum of squares	Mean square	*F*-value	*p*-value
*Y*_1_	Model	14576.52	1619.61	18.96	0.0170*
	*A*	3160.12	3160.12	37.00	0.0089
	*B*	1984.50	1984.50	23.23	0.0170
	*C*	703.13	703.13	8.23	0.0641
	*AB*	2756.25	2756.25	32.27	0.0108
	*AC*	1764.00	1764.00	20.65	0.0200
	*BC*	420.25	420.25	4.92	0.1132
	*A* ^2^	3344.14	3344.14	39.15	0.0082
	*B* ^2^	1316.57	1316.57	15.41	0.0294
	*C* ^2^	1824.14	1824.14	21.36	0.0191
	Residual	256.25	85.42		
	Cor total^#^	14832.77			
*Y*_2_	Model	0.0184	0.0020	13.39	0.0279*
	*A*	0.0021	0.0021	13.66	0.0344
	*B*	0.0015	0.0015	9.75	0.0523
	*C*	0.0076	0.0076	49.68	0.0059
	*AB*	0.0007	0.0007	4.27	0.1307
	*AC*	0.0004	0.0004	2.63	0.2035
	*BC*	0.0003	0.0003	2.13	0.2407
	*A* ^2^	0.0001	0.0001	0.3943	0.5746
	*B* ^2^	0.0043	0.0043	27.92	0.0132
	*C* ^2^	0.0025	0.0025	16.72	0.0264
	Residual	0.0005	0.0002		
	Cor total^#^	0.0188			
*Y*_3_	Model	511.74	56.86	9.91	0.0425*
	*A*	185.28	185.28	32.28	0.0108
	*B*	108.49	108.49	18.90	0.0225
	*C*	8.74	8.74	1.52	0.3051
	*AB*	17.72	17.72	3.09	0.1771
	*AC*	0.3969	0.3969	0.0692	0.8096
	*BC*	0.3721	0.3721	0.0648	0.8155
	*A* ^2^	74.46	74.46	12.97	0.0367
	*B* ^2^	149.32	149.32	26.02	0.0146
	*C* ^2^	115.79	115.79	20.18	0.0206
	Residual	17.22	5.74		
	Cor total^#^	528.96			

*significant

^#^total sum of squares

PDI (Polydispersity index) indicates the homogeneity distribution of the size population in a sample. The system is considered homogenous if the PDI value is less than 0.3 [27,36]. The PDI range of all formulations was found to be from 0.151 to 0.288, which indicated that the formulations were found to be homogenous. The quadratic equation (Equation 2) showed that the oil and surfactant have a negative effect on the PDI, while sonication time has a positive effect.

Reports suggest that a high concentration of surfactant and oil can result in the production of homogenous nanoparticles. The high sonication time can enhance PDI because of the heterogeneity of the emulsion [37,38]. Turmeric oil (*A*) (*p*-value of 0.0344 and *F*-value of 13.66) and sonication time (*C*) (*p*-value of 0.0059 and F-value of 49.68) had a significant effect on PDI (*Y*_2_). Tween 80 had no significant effect on the Y_2_ variable (Figure 2). In addition, the combination of all independent factors did not have any significant influence on the *Y*_2_ variable (Table 3).

**Figure 4.**
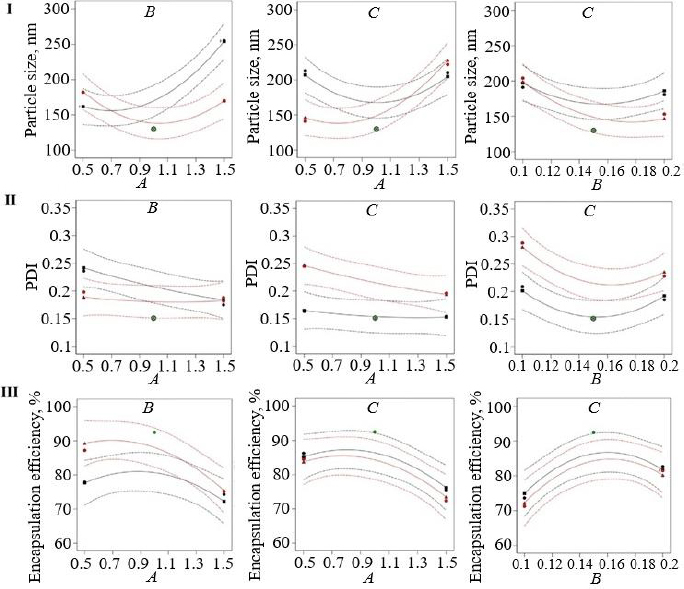
Interaction plots between all independent factors affecting PS (I), PDI (II) and EE of curcumin (III)

The EE of curcumin-loaded nanoemulsion formulations was found within a range from 71.15±2.5 % to 92.45±2.4 %. Turmeric oil has a negative impact, and Tween 80 positively impacts curcumin encapsulation (Equation 3). In addition, sonication time showed a non-significant effect on the EE. Figure 3 showed a negative magnitude of oil concentration could increase the EE of curcumin due to the low flowing rate. Tween 80 concentration can decrease interfacial tension and increase the solubility of curcumin due to the high HLB value that enhances the EE [39]. Turmeric oil (*A*) (F-value 32.28) and Tween 80 (*B*) (*F*-value 18.90) had a significant effect (*P* < 0.05) on the EE of curcumin (*Y*_3_). However, the sonication time and all the combinational independent variables did not have any significant effect on the *Y*_3_ independent variable (Table 3 and Figure 3). The coefficient of variation was chosen to confirm the precision and robustness of the model. A good degree of agreement was observed between predicted and actual values, showing that the established model is valid (Table 4). Also, the probable interaction between all the independent variables was examined, and a strong effect was observed on the dependent variables (Figure 4).

**Table 4.** Actual and predicted values for formulation CNE_1_ to CNE_13_.

Formulation	Particle size,nm		Polydispersity index		Encapsulation efficiency, %	
	Actual	Predicted	Actual	Predicted	Actual	Predicted
CNE_1_	153.0	146.87	0.228	0.235	81.49	80.19
CNE_2_	162.7	161.87	0.236	0.241	77.91	77.68
CNE_3_	141.9	146.25	0.245	0.252	84.46	83.70
CNE_4_	170.8	176.12	0.188	0.182	75.20	75.42
CNE_5_	222.2	228.00	0.196	0.192	72.38	73.45
CNE_6_	255.4	259.24	0.175	0.184	74.32	72.27
CNE_7_	130.6	129.40	0.151	0.149	92.45	92.45
CNE_8_	191.4	197.12	0.209	0.201	73.62	74.91
CNE_9_	213.3	207.00	0.163	0.168	86.24	85.16
CNE_10_	182.2	189.80	0.198	0.189	87.21	89.26
CNE_11_	210.8	204.50	0.154	0.157	75.42	76.17
CNE_12_	181.9	186.10	0.185	0.192	82.65	81.67
CNE_13_	204.8	198.80	0.288	0.280	71.24	72.21

### Characterization of nanoemulsion

#### Particle size and polydispersity index

**Figure 5.**
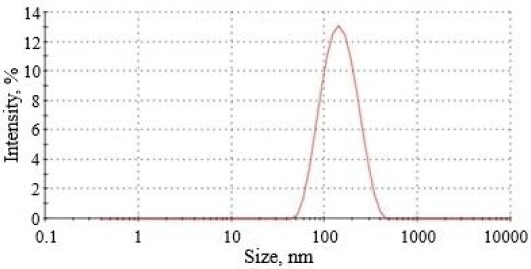
Result of PS and PDI analysis of optimized formulation (formulation CNE_7_)

Curcumin-loaded nanoemulsion had PS between 130.6 and 255.4 nm with a PDI of 0.151 to 0.288. The optimized formulation (CNE_7_) depicted a PS of 130.6 nm with a PDI of 0.151 (Figure 5). The PS imparts a strong impression on lung deposition patterns and total bioavailability. The optimized formulation could improve pulmonary drug delivery due to its small PS, facilitating efficient deposition within the lower respiratory tract. In addition, the narrow PDI observed in the optimized formulation indicated a uniform size distribution of particles within a nanoemulsion without aggregation [40,41].

#### Nanoparticle tracking analysis

**Figure 6.**
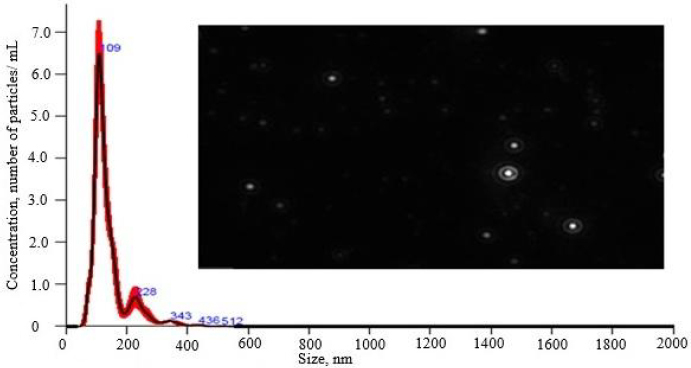
Results of nanoparticles tracking analysis of optimized nanoemulsion formulation (CNE_7_)

NTA can provide more specific information on PS and concentration. It also provides a size distribution peak. However, DLS cannot provide concentration information of the particles. In the NTA test, the optimized formulation had a mean PS of 138.3±1.6 nm and a total particle concentration of 3.78×10^12^ particles/mL (Figure 6). The mean PS values obtained from NTA were found to be closer to the optimized nanoemulsion DLS data, which signified confirmation of the PS [42].

#### Zeta potential

**Figure 7.**
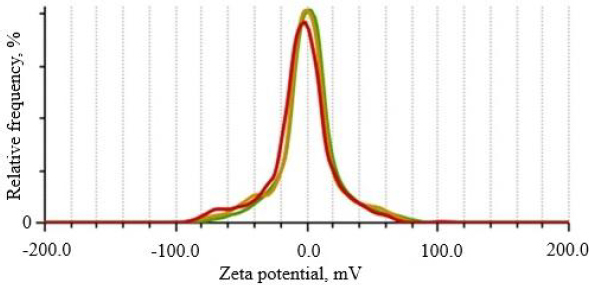
Result of zeta potential analysis of optimized formulation (formulation CNE_7_).

The zeta potential of the optimized curcumin-loaded nanoemulsion (CNE_7_) was found to be -1.7 ± 0.6 mV, which indicated a slightly negative or near-neutral charge (Figure 7). This can be due to the use of non-ionic surfactants, Tween 80, which does not impart a strong surface charge. Researchers have reported similar findings, demonstrating sufficient colloidal stability and compatibility with biological systems in nanoemulsions with neutral zeta potential [43]. Nanoemulsions with neutral zeta potential demonstrate better biocompatibility since they avoid unwanted reactions with negatively charged cell membranes, while strongly charged systems cause higher toxicity or poor cellular uptake. These systems demonstrate effective biological membrane penetration capabilities, which improve drug delivery while maintaining low toxicity levels. Furthermore, studies have demonstrated that these systems effectively penetrate biological membranes, enhancing drug delivery without causing significant toxicity [43]. The near-neutral charge of the optimized formulation showed stability and has the potential for effective pulmonary drug delivery. Similarly, non-ionic surfactant-based nanoemulsions have been reported for excellent bioavailability and safety in animal models [44].

#### Encapsulation efficiency and drug loading

The EE of all the curcumin-loaded nanoemulsions was found to be between 71.24±2.5 and 92.45±2.4 %. The optimized formulation showed an EE of 92.45±2.4 % and DL of 2.8±1.5 %. This high EE was achieved because of the presence of Tween 80 in nanoemulsion. The Tween 80 can rapidly adsorb onto particle surfaces and decrease the interfacial tension within the nanoemulsion. In addition, it has been reported that a high concentration of Tween 80 improved curcumin encapsulation in emulsion. Apart from the effect of Tween 80 concentration, the solubility of curcumin in the oil phase also helps achieve a high EE [26,45,46].

#### Morphology analysis

**Figure 8.**
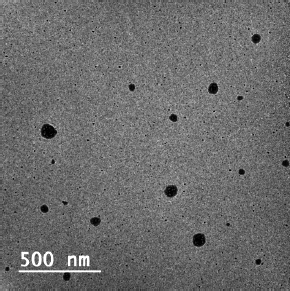
TEM image of optimized nanoemulsion (formulation CNE_7_)

The optimized nanoemulsion formulation has spherical particles with a uniform appearance and no sign of aggregation in TEM analysis (Figure 8). The size of particles observed in the TEM image was found to be close to the results obtained in the DLS analysis. However, TEM and DLS do not have any correlation due to their different working principles. The DLS works on the Brownian motion and intensity-based approach, while the TEM image is developed because electron flux passes via the sample and the number-based approach. In addition, the DLS represents hydrodynamic diameter, and the TEM represents the particle's surface area based on their projected view [27].

#### Antioxidant activity

The difference between the oxidant and antioxidant presence in the body leads to oxidative stress and contributes to the development of ROS. The body is adversely affected by the stress produced by ROS, which is a significant factor in autophagy, regeneration, and apoptosis [47]. In addition, ROS increases pulmonary edema and tissue damage due to the upregulation of pro-inflammatory cytokines in ALI [48,49]. Curcumin has the potential to inhibit ROS, which leads to an increment of the antioxidant defense system [50]. It also enhanced superoxide dismutase, catalase, and glutathione peroxidase levels [51,52]. The ascorbic acid was chosen as a reference compound with an *IC*_50_ = 17 ± 0.04 μg/mL. The optimized curcumin nanoemulsion showed an *IC*_50_ = 25.65 μg/mL, which indicated that the nanoemulsion has powerful free radical scavenging properties and can alleviate inflammation [53].

#### Reactive nitrogen species inhibition (RNI assay)

The upregulation of pro-inflammatory mediators is a key feature of ALI, which involves an increment in nitric oxide synthase expression responsible for nitric oxide production. The balance between NO/ROS plays a crucial role in normal vascular functioning, inflammation, and immune response. In addition, excessive ROS in combination with NO leads to the production of peroxynitrite, which enhances vessel permeability and damages blood vessels and lipid membranes [54,55]. Reports indicate that curcumin can inhibit nitric oxide synthase activity and potentially regulate surfactant expression in lung tissues [16]. The optimized formulation had an *IC*_50_ value of 21.76 μg/mL for NO inhibition. These results suggested that formulation could be used to counter oxidative stress induced by lowering reactive nitrogen species in lung cancer cells, which are associated with inflammation and pathological processes [53].

#### Stability studies

**Figure 9.**
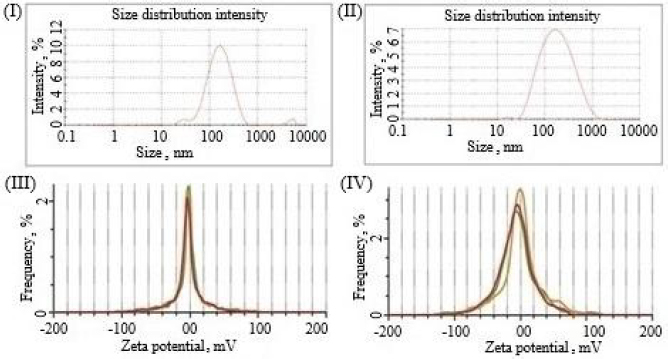
Results of PS distribution (I and II) and zeta potential (III and IV) of the optimized formulation at 4±1 °C and 25±2 °C, respectively, with 60±5 % RH for 3 months

The optimized formulation did not show any major change in physical appearance and size distribution at 4 °C (Figure 9I) and 25 °C (Figure 9II). In addition, the optimized formulation showed *ζ* potential - 2.9±0.1 at 4±1 °C (Figure 9III) and -1.4±0.3 at 25±2 °C / 60±5 % RH (Figure 9IV) for 3 months with a PDI value of 0.285 and 0.365, respectively. This minimal change in size distribution and zeta potential indicated the stability of the nanoemulsion [42].

### In vivo studies

**Figure 10.**
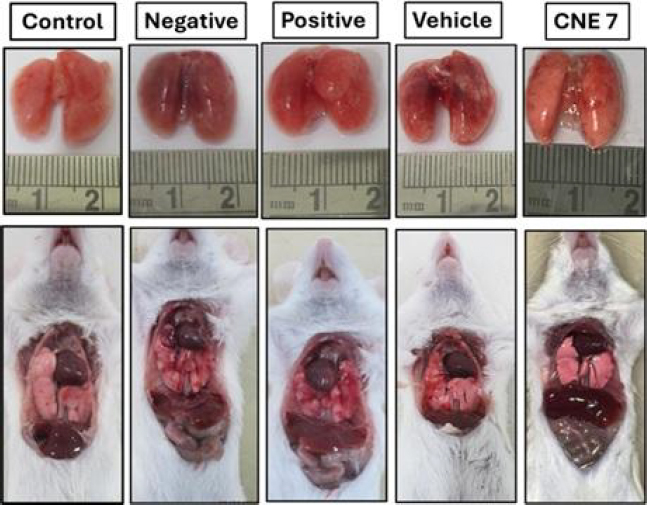
Comparative images of harvested lungs from different experimental groups (control, negative control, positive control, vehicle and optimized formulation group CNE_7_)

We successfully developed the LPS-induced ALI/ARDS model in the BALB/c mice and evaluated the efficacy of the optimized formulation (formulation CNE_7_). LPS causes alveolar wall thickening, inflammatory cell infiltration, and lung pulmonary edema. The animals were sacrificed from each group at 24-hour and 48-hour time points. The bronchoalveolar lavage fluid (BALF) and cytokine estimation were performed at 24 hours, and histological analysis was performed at 48 hours [10]. The control and positive groups (standard treatment) showed no damage in the harvested lungs. However, the negative group and vehicle group depicted hemorrhage and edema in the lung. The optimized formulation-treated group showed no lung hemorrhage (Figure 10).

#### BALF count / BAL fluid inflammatory cell count

**Figure 11.**
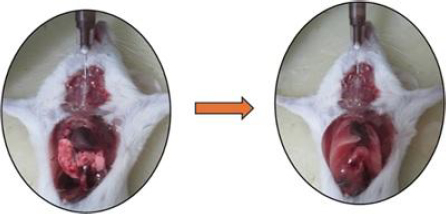
Representative image of bronchoalveolar lavage fluid (BALF) collection from dissected mice

The BALF total cell count is used to evaluate the degree of inflammation and cellular infiltration in the LPS-induced ALI/ARDS model. The euthanized mice were injected with 1 mL of PBS via trachea for efficient retrieval of BALF (Figure 11).

**Figure 12.**
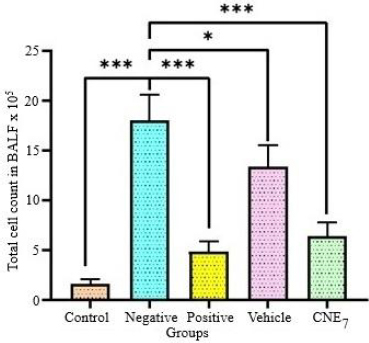
Effect of optimized formulation on inflammatory cell recruitment. The results are shown in mean ± SEM. Statistical analysis was done using One-Way ANOVA followed by Tukey’s test at *p*-values of *0.033 and *** <0.001

In the control group, the total BALF cell count was minimal, representing the normal physiological baseline for control group mice. In contrast, the negative control group showed a significant increase in total cell count (*p* < 0.001), which is due to the infiltration of the immune cells, such as macrophages and neutrophils, due to inflammation, which is common in ALI/ARDS [56,57]. The positive control group (dexamethasone) showed a significant reduction in total cell count compared to the negative control group (*p* < 0.001). Dexamethasone suppresses pro-inflammatory cytokines by inhibiting the recruitment of neutrophils and other inflammatory cells. This mechanism effectively lowers the number of inflammatory cells that enter the alveolar space, resulting in fewer cells in the BALF [58]. Also, no significant difference was observed between the positive control and control groups (*p* = 0.21). The optimized formulation-treated group significantly decreased the BALF total cell count compared to the negative control group (*p* < 0.001). The reduction was slightly less pronounced than the positive control group (*p* = 0.77). The therapeutic effect of the optimized formulation remained statistically significant compared to the vehicle-treated group (*p* = 0.004) (Figure 12).

#### Histopathological changes

**Figure 13.**
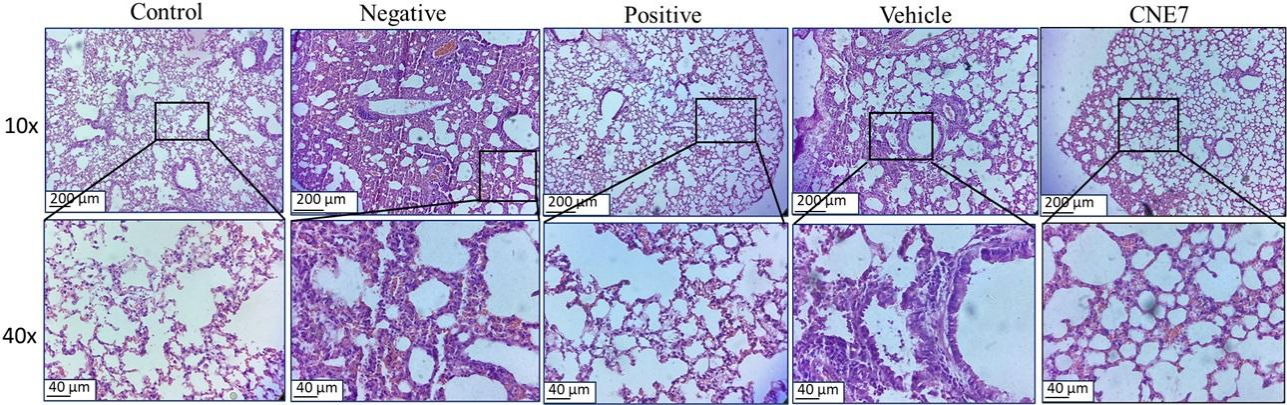
Histopathological features of hematoxylin and eosin-stained lungs during efficacy studies on LPS-induced ALI/ARDS mice model

Histological analysis of H&E-stained lung tissue sections revealed differences between the group treated with the optimized formulation and the negative control group. The analysis was conducted at the 48-hour time point, as lung damage is most prominent between 48 and 96 hours post-LPS induction. Similar results are reported earlier [10]. The control group exhibited a healthy lung structure characterized by intact alveolar spaces and thin alveolar walls, with no evidence of inflammatory cell infiltration. The negative control group showed significant pathological changes, including substantial infiltration of inflammatory cells, thickening of the alveolar walls, and the onset of interstitial edema. The findings indicate ALI, consistent with previous research on LPS-induced ALI/ARDS models [59]. The optimized formulation-treated group depicted alleviation of the histopathological changes, such as reduced alveolar wall thickening and minimal inflammatory cell infiltration, compared to the negative control group. These improvements aligned with the ELISA results, which demonstrated a reduction in BALF's pro-inflammatory cytokine levels (TNF-α and IL-1β) (Figure 13).

This indicates that optimized formulation effectively modulates the inflammatory response and maintains lung architecture. The vehicle-treated group showed slight advantages compared to the negative control through a moderate reduction in both alveolar wall thickening and inflammatory cell counts. This suggested that the vehicle formulation offers limited protection, which may be due to the anti-inflammatory properties of turmeric oil [60]. However, the protective effect of the vehicle group was significantly less pronounced than those observed with the optimized formulation, which exhibited combined anti-inflammatory and antioxidant properties.

#### Inflammatory cytokine levels

**Figure 14.**
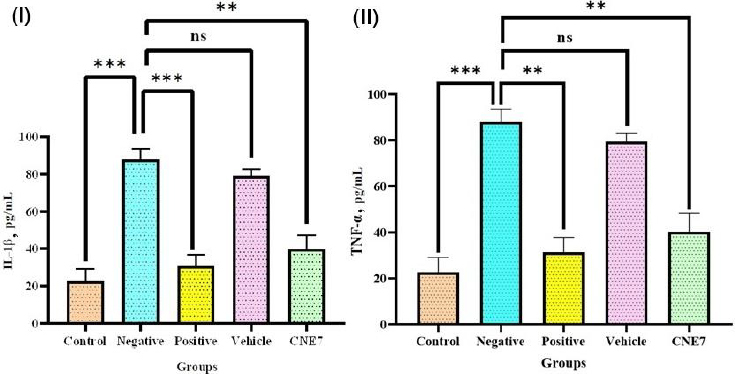
Results of ELISA assay to determine (I) IL-1β and (II) TNF-α levels in ALI/ARDS BALB/c mice treated with the optimized formulation (mean ± SEM, *n* = 3). Statistical analysis was done using One-Way ANOVA followed by Tukey’s test at *p*-values of ^ns^0.12, *0.033, **0.002 and ***<0.001

Cytokines play an important role in the pathogenesis of ALI/ARDS. IL-1β and TNF-α are the key pro-inflammatory cytokines elevated in ALI/ARDS conditions. The ELISA analysis was performed on the BAL fluid supernatant to determine the therapeutic efficacy of the optimized formulation (formulation CNE_7_). The control group represented a baseline of TNF-α and IL-1β levels, which showed no inflammation (Figure 14). In contrast, the negative control group showed a significant (*p* < 0.001) enhancement in TNF-α and IL-1β levels due to LPS-induced inflammation. This confirmed the successful development of an ALI/ARDS model [10]. Optimized formulation significantly reduced IL-1β and TNF-α levels compared to the negative control (*p* = 0.002 and p = 0.003), confirming its anti-inflammatory potential. This reduction can be due to the ability of curcumin to inhibit pro-inflammatory pathways like NF-κB signaling. NF-κB is a key regulator of inflammation that controls the expression of other cytokines like TNF-α and IL-1β, chemokines, and enzymes like COX-2 and iNOS [61]. No significant difference was recorded between the optimized formulation treated group and the positive control group (*p* = 0.63 for TNF-α and *p* = 0.57 for IL-1β), which suggested that the formulation provides therapeutic effects comparable to positive control, *i.e*., standard treatments. The vehicle-treated group lowered TNF-α and IL-1β levels more than the negative control (*p* = 0.66 and *p* = 0.61) but remained higher than the optimized formulation-treated group. These outcomes indicated that the vehicle has anti-inflammatory effects due to the presence of the turmeric oil component [60].

The therapeutic outcomes of this study are supported by a previous study reported by Toden et al. Turmeric oil enhances the biological activity of curcumin and improves its bioavailability, which enhances its anti-inflammatory effects [62]. These findings were aligned with the histological results, where optimized formulation effectively preserved lung architecture and reduced inflammatory infiltrates. Overall, these results showed that the optimized formulation significantly lowered levels of pro-inflammatory cytokines in ALI/ARDS mouse model.

## Conclusions

The optimized curcumin-loaded turmeric oil-based nanoemulsion demonstrated significant potential in mitigating inflammation and oxidative stress. Its enhanced stability, improved encapsulation efficiency, and effective modulation of pro-inflammatory cytokines suggest its suitability for therapeutic applications. The formulation exhibited promising *in vitro* and *in vivo* outcomes, indicating its potential for treating ALI/ARDS. These findings support further exploration and clinical translation of nanoemulsion-based delivery systems for inflammatory lung diseases.
